# Using historical woodland creation to construct a long‐term, large‐scale natural experiment: the WrEN project

**DOI:** 10.1002/ece3.2066

**Published:** 2016-03-30

**Authors:** Kevin Watts, Elisa Fuentes‐Montemayor, Nicholas A. Macgregor, Victor Peredo‐Alvarez, Mark Ferryman, Chloe Bellamy, Nigel Brown, Kirsty J. Park

**Affiliations:** ^1^ Forest ResearchAlice Holt LodgeFarnhamSurreyGU10 4LHUK; ^2^ Biological and Environmental SciencesSchool of Natural SciencesUniversity of StirlingStirlingFK9 4LAUK; ^3^ Natural EnglandNobel House, 17 Smith SquareLondonSW1P 3JRUK; ^4^ Durrell Institute of Conservation and Ecology (DICE)School of Anthropology and ConservationUniversity of KentCanterburyKent CT2 7NRUK; ^5^ Forest ResearchNorthern Research StationRoslinMidlothianEH25 9SYUK; ^6^ Natural EnglandUnex HousePeterboroughPE1 1NGUK

**Keywords:** Ecological networks, landscape‐scale conservation, natural experiment, woodland creation, WrEN project

## Abstract

Natural experiments have been proposed as a way of complementing manipulative experiments to improve ecological understanding and guide management. There is a pressing need for evidence from such studies to inform a shift to landscape‐scale conservation, including the design of ecological networks. Although this shift has been widely embraced by conservation communities worldwide, the empirical evidence is limited and equivocal, and may be limiting effective conservation. We present principles for well‐designed natural experiments to inform landscape‐scale conservation and outline how they are being applied in the WrEN project, which is studying the effects of 160 years of woodland creation on biodiversity in UK landscapes. We describe the study areas and outline the systematic process used to select suitable historical woodland creation sites based on key site‐ and landscape‐scale variables – including size, age, and proximity to other woodland. We present the results of an analysis to explore variation in these variables across sites to test their suitability as a basis for a natural experiment. Our results confirm that this landscape satisfies the principles we have identified and provides an ideal study system for a long‐term, large‐scale natural experiment to explore how woodland biodiversity is affected by different site and landscape attributes. The WrEN sites are now being surveyed for a wide selection of species that are likely to respond differently to site‐ and landscape‐scale attributes and at different spatial and temporal scales. The results from WrEN will help develop detailed recommendations to guide landscape‐scale conservation, including the design of ecological networks. We also believe that the approach presented demonstrates the wider utility of well‐designed natural experiments to improve our understanding of ecological systems and inform policy and practice.

## Introduction

### Large‐scale experimentation in applied ecology and conservation

While the importance of experimentation to advance ecology and inform conservation is widely acknowledged, it remains a relatively rare approach at larger spatial scales (Debinski and Holt 2000; Ewers et al. 2011; Haddad 2012). There are two fundamental challenges to large‐scale experimentation in ecology. The first is a trade‐off between the spatial scale necessary to ensure ecological realism and obtain evidence applicable to practical conservation and the ability to exert experimental control and replication (Debinski and Holt 2000; Haddad 2012). The second challenge is the difficulty, and cost, of running experiments over the temporal scales necessary to detect effects, given the time it often takes biodiversity to respond to landscape change. These temporal lags are linked to extinction debt, for instance as a consequence of habitat fragmentation, and to immigration credit as a consequence of the delay between habitat creation and species colonization (Tilman et al. 1994; Jackson and Sax 2010). Extinction lags may be fairly short for species that respond quite rapidly to habitat loss and fragmentation, but other species may persist long after fragmentation events. Similarly, colonization lags may be fairly short for mobile species in well‐connected early successional ecosystems (e.g., grasslands), whereas poorly dispersing species may take a very long time to colonize slowly‐developing ecosystems (e.g., forests) in fragmented landscapes (e.g., De Frenne et al. 2011). The balance between these spatial and temporal components collectively determines the biodiversity within a landscape. These are also the driving forces behind the theory of island biogeography, which has influenced conservation policy and practice for many decades (MacArthur and Wilson 1967; Diamond 1975; Simberloff and Abele 1976).

There have been several notable advances in landscape‐scale experimentation that have begun to overcome some of these spatial and temporal challenges. For example, the Biological Dynamics of Forest Fragments Project (BDFFP), established in the Brazilian Amazon in 1970s (Lovejoy and Oren 1981), adopted a large‐scale experimental approach to examine the impacts of fragmentation on biodiversity through the creation of a replicated sequence of fragmented patches ranging from 1 to 100 ha. However, this study was primarily focused on the size of forest fragments (a patch or site‐level attribute) in an attempt to define critical fragment size (Lovejoy and Oren 1981). Subsequent studies have incorporated the role of patch isolation (a landscape‐level attribute) along with patch area. For instance, the Savannah River Site (SRS) project in South Carolina, which has been established for over 25 years, developed a large‐scale, replicated experiment, in which 27 equal‐sized (1.64 ha) patches of open longleaf pine savanna were created within a large plantation forest (Haddad 1997). Patches varied in whether or not they were connected to another patch by a corridor and in their distance (ranging between 64 and 384 m) from other patches (Haddad 1999). This project aims to test the ecological consequences of corridors as a strategy to combat habitat loss and fragmentation (Haddad 1997, 1999, 2012).

Other studies attempt to consider a range of both site‐ and landscape‐level attributes, including the amount of surrounding habitat and the nature of the surrounding matrix. The recently established Stability of Altered Forest Ecosystems (SAFE) project in the lowland tropical forests of Borneo is one such example (Ewers et al. 2011). This forest fragmentation experiment, which is embedded within the planned conversion of native rainforest to oil palm plantation, has adopted a robust and sophisticated hierarchical, fractal sampling design. This design is intended to allow for the discrimination of patch and landscape‐level effects, including the influence of the surrounding matrix, while still maintaining a high level of replication (Ewers et al. 2011). The level of replication and experimental control in large‐scale experiments has recently been advanced by the Metatron project, albeit at the expense of spatial scale (Legrand et al. 2012). The Metatron, established in France, provides a robust experimental design for the study of “meta‐systems”; it consists of 48 (10 × 10 m) enclosed “patches” (enclosed greenhouses) interconnected by corridors that can be opened or closed. In addition, environmental conditions, including temperature, light intensity, precipitation, and humidity, can be controlled independently within each patch. These manipulative landscape‐scale experiments, and many others, have fundamentally improved ecological understanding of habitat loss and fragmentation and provided many guiding principles for conservation.

### New challenges from landscape‐scale conservation

The need for experimental studies at large spatial and temporal scales is increasing as a result of the current shift toward landscape‐scale conservation, which has been widely embraced by conservation communities worldwide (e.g., Boitani et al. 2007; Warboys et al. 2010; Fitzsimons et al. 2013). This approach is embedded in conservation policy in the United Kingdom (UK) and has resulted in the initiation of various landscape‐scale schemes (Macgregor et al. 2012). A prominent aspect of this approach to conservation is the concept of ecological networks, defined as a spatial network of core habitat areas, corridors, stepping stones and buffer zones with the aim of maintaining the functioning of ecosystems and increasing the persistence and movement of species across fragmented landscapes (Bennett and Wit 2001; Jongman and Pungetti 2004; Bennett and Mulongoy 2006; Lawton et al. 2010).

The basic concepts behind landscape‐scale conservation and ecological networks are appealing and based on sound ecological principles (see Fischer and Lindenmayer 2007; Fahrig 2003; SLOSS principles of Diamond 1975). However, the simple and logical principles that have been put forward to guide policy and practice (e.g., Lawton et al. 2010) encompass a potentially wide and complex range of site‐ and landscape‐level actions that are not necessarily compatible or achievable in practice. Here, the empirical evidence is limited and equivocal (Boitani et al. 2007; Humphrey et al. 2015). There is an ongoing debate within the scientific and conservation communities on the relative merit of, and balance between, site‐ and landscape‐level actions to conserve biodiversity within fragmented landscapes. Some authors have promoted site‐based actions to increase habitat amount regardless of spatial configuration (Fahrig 2013), to balance habitat area, isolation, and configuration (Prugh et al. 2009; Hanski 2015) or to increase habitat quality (Moilanen and Hanski 1998; Hodgson et al. 2009, 2011). Others focus on the merits of landscape‐level actions to improve connectivity (Doerr et al. 2011) through the creation of corridors (Beier and Noss 1998; Haddad 1999) and actions to improve the surrounding matrix (Baum et al. 2004; Eycott et al. 2012). Some have argued that ecological networks are based on oversimplifications of complex ecological concepts and offer little for biodiversity conservation beyond a simple conceptual framework, which may be misdirecting limited resources (Boitani et al. 2007).

This makes it hard to draw conclusions about the relative importance of the individual and combined effects of the different components of landscape‐scale conservation on a broad suite of species. Prioritizing conservation actions at either local or landscape scale can therefore sometimes be more a matter of faith and tradition than evidence‐based practice (e.g., Sutherland et al. 2004). A greater use of experimental approaches could clearly help to resolve this situation, increasing the chances of teasing apart the relative influence on biodiversity of different attributes of sites and landscapes (and thus of different management actions that might be considered as part of landscape‐scale conservation). But the time and resource implications of carrying out experimental manipulations of whole landscapes over the time periods required are considerable.

### The role of natural experiments

Well‐designed natural experiments have the potential to overcome some of the challenges outlined above and provide much‐needed evidence to inform current and future conservation action (McGarigal and Cushman 2002) required to meet international commitments to halting declines in biodiversity. Rather than carrying out direct experimental manipulation of a site or landscape, natural experiments overlay an experimental design on an ecosystem where change or active manipulation has occurred or is planned, beyond the control of the researcher (Diamond 1986; Carpenter et al. 1995). As such, they fall between true manipulative experiments and the more common, but less rigorous, correlative or observational studies (Diamond 1986; Lindenmayer 2009). They usually occur at larger scales than true experiments, often increasing the chances of obtaining results with direct application to conservation management. In addition, natural experiments can also overcome pragmatic issues of land tenure/control, funding, and urgency for evidence, while still, if well‐designed, maintaining a degree of experimental control and producing robust evidence.

The Tumut Fragmentation Study in NSW in southeastern Australia is a good example of a well‐designed long‐term, large‐scale natural experiment (Lindenmayer 2009). The study focuses on a 50,000‐ha pine plantation which was established by clearing native eucalyptus forest between 1932 and 1985. When the plantation was created, isolated patches of native forest were left within the non‐native pine forest. Eighty‐six of these native woodland patches were selected by a stratified random sample, based on patch size, time since isolation, and dominant tree species (Lindenmayer 2009). A range of taxa have been surveyed in these patches and also in comparable sites in the pine plantation and in large contiguous areas of native forest nearby. This enables conclusions to be drawn about how the presence and abundance of different species are affected by different degrees of fragmentation of native vegetation and its replacement with the non‐native plantation forest.

There are many landscape‐scale conservation projects throughout the world (Bennett and Mulongoy 2006; Warboys et al. 2010; Macgregor et al. 2012; Fitzsimons et al. 2013), which offer great potential for studying the effects of site‐ and landscape‐scale conservation on biodiversity. However, most are established in an ad hoc manner and, as yet, lack the experimental control and timescale necessary to form the basis of a robust natural experiment to yield evidence to inform landscape‐scale conservation.

### Principles for the design of natural experiments to inform landscape‐scale conservation

A key feature of landscape‐scale conservation, particularly in areas that have experienced major land clearing, is the restoration and creation of habitats to create new conservation sites, expand or buffer existing ones, and create stepping stones and corridors in an attempt to reconnect habitat fragments (i.e., the development of “ecological networks”; e.g., Jongman and Pungetti 2004). For any natural experimental study aiming to inform this approach to conservation, we propose five principles that should be followed (building on suggestions made by McGarigal and Cushman 2002):

1. Focus on investigating the effects of habitat restoration and creation, rather than (or in addition to) habitat removal and fragmentation. Much of the existing evidence is drawn from fragmentation studies, and there is little evidence to show that the ecological consequences of removing natural land cover (i.e., fragmentation) and the benefits of putting it back (i.e., creation) are reciprocal;
2. Study real landscapes at sufficiently large spatial scales to ensure ecological realism and the applicability of evidence;
3. Incorporate appropriately long timescales to account for the considerable lag in ecosystem development and colonization associated with habitat restoration and creation;
4. Sample a wide range of explanatory site‐ and landscape‐level variables to understand their relative and combined impacts. Relatively few past studies have examined the relative importance of the full range of relevant variables (Humphrey et al. 2015);
5. Examine the response of a wide range of taxa in order to identify potential important differences in the requirements of different taxonomic or functional groups, as well as to attempt to draw out general recommendations.

Using these principles, researchers could design studies to investigate the effects of expected future landscape changes; however, this approach faces the temporal challenge mentioned above. Alternatively, there are potential opportunities to learn from the past. Studies of this sort could be conducted in a range of landscapes and ecosystems where two conditions are met. First, past restoration or creation of natural vegetation or other land cover needs to have occurred over sufficiently large areas and periods of time. Second, this needs to have been recorded sufficiently well to be able to accurately map changes in land cover through time.

### Research opportunities from past changes in forest cover

Temperate forests and woodlands – where conservation of biodiversity would greatly benefit from better information to inform design of ecological networks – potentially provide study landscapes that satisfy these two conditions. Forest cover worldwide, including in many temperate areas, has undergone substantial decreases in the past 300 years (Ramankutty and Foley 1999). However, in parts of Europe, North America and Australia this loss is being reversed by natural expansion and forest restoration and creation (Kleijn and Sutherland 2003; FAO, 2010; Keenan et al. 2015).

In the UK, a similar and possibly even more extreme pattern of change in woodland cover has occurred (“woodland” is the term commonly used in the UK to describe any forested area; for convenience, we use this term hereafter in the paper). At the beginning of the 20th century, woodland was estimated to cover less than 5% of the land area of the UK (Mason 2007). Since then, as a result of woodland creation that had started in the middle of the 19th century and accelerated over the 20th century, woodland cover has increased to approximately 13% of UK land (Harmer et al. 2015). This long program of woodland creation within the UK has led to the development of landscapes containing a large number of woodland patches of varying age, size, and levels of isolation. Many of these new woodlands were established on former agricultural land, without remnant woodland biodiversity. Therefore, the presence of species within these new woodlands represents successful colonization, presumably mediated by attributes of the woodland sites and the landscapes around them. Accurate maps of major features in UK landscapes are available over a series of time intervals from 1840s, enabling many of these changes in woodland cover to be potentially identified and dated.

### The WrEN project: Woodland Creation and Ecological Networks

The WrEN project (www.tinyurl.com/wren-project) is a natural experiment that is taking advantage of the opportunities for ecological research offered by the UK woodland landscapes outlined above. The project, which was set up in 2013, aims to explore the potential for a natural experimental approach to study biodiversity in fragmented landscapes, with the specific goal of informing conservation of woodland biodiversity both in the UK and elsewhere.

In this paper, we describe the development of the WrEN project. We outline the landscapes that were chosen as study areas, the process developed to determine the key site and landscape variables to be studied, and how these were used to select a large number of sites within the study landscapes. We present results on the main attributes driving variation in woodland patches at both the local and landscape levels. In particular, we focus on answering a question that was crucial to the design and establishment of the project: Do the woodland landscapes available in the UK provide a study system that satisfactorily addresses the five principles we have outlined above for a robust natural experiment to inform landscape‐scale conservation?

## Methods

**Figure 1:**
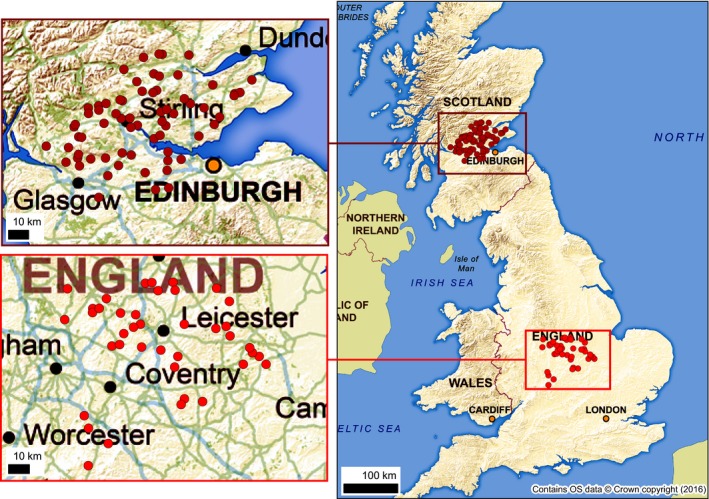
Location of woodland study sites in the Scotland (*n* = 67) and England (*n* = 39) WrEN study landscapes, showing location of these study landscapes in the UK.

Two study landscapes were selected for the study, one in Scotland (~7335 km^2^) and the other in England (~8570 km^2^ (Fig. 1). Both areas are dominated (>70%) by agricultural land and represent typical lowland landscapes in the UK. The focus on fairly homogeneous lowland agricultural landscapes ensured that (i) other covariates that would otherwise influence the study (e.g., topography, climate and soil types) were minimized and (ii) that there were almost certainly no remnant woodland species within these agriculturally dominated sites prior to woodland creation (a key element of the study design). These landscapes are also typical of those in which much conservation action is targeted, so maximizing the potential to produce results with direct practical application. The landscape in Scotland was selected owing to the authors’ previous knowledge of the area and the availability of suitable sites that had been previously visited and surveyed for woodland biodiversity (e.g., Fuentes‐Montemayor et al. 2012, 2013). The England study landscape was focussed around the National Forest, a landscape that has experienced considerable woodland creation over the past 25 years (Harmer et al. 2015). This landscape is also more intensively farmed with a higher percentage of agricultural land and lower and more fragmented cover of woodlands, broadening the range of site variables included in the study.

To enable the establishment of a robust natural experiment, we used a systematic process to select suitable woodland sites within the two study landscapes. This process was informed by a comprehensive literature review, conducted as the first step of the project (see Humphrey et al. 2015). The review identified the variables that can influence biodiversity within fragmented forest landscapes and reviewed the existing evidence base. The review identified three patch or site‐level variables: (i) *patch area/size*; (ii) *patch characteristics/quality*, equating to the measures of within‐patch configuration, structure, or composition, for example, tree or plant species richness, tree size; and (iii) *site age/ecological continuity*, the length of time tree cover has been present (ecological continuity can differ from tree age as a consequence of ecological succession or management). Three landscape‐level variables were also identified: (iv) *amount of surrounding habitat*, measured as the amount, extent, or proportion of similar vegetation surrounding each target patch; (v) *degree of spatial isolation*, usually defined as a distance or connectivity measure to the nearest similar habitat patch; and (vi) *nature of the surrounding matrix*, the amount, extent, or proportion of different land‐uses surrounding a target habitat patch, for example, percentage cover of agricultural or urban land. The review also revealed the general paucity of evidence, with only 4 out of 104 studies examining all six factors simultaneously, and only 29 examining five or more (Humphrey et al. 2015).

**Figure 2:**
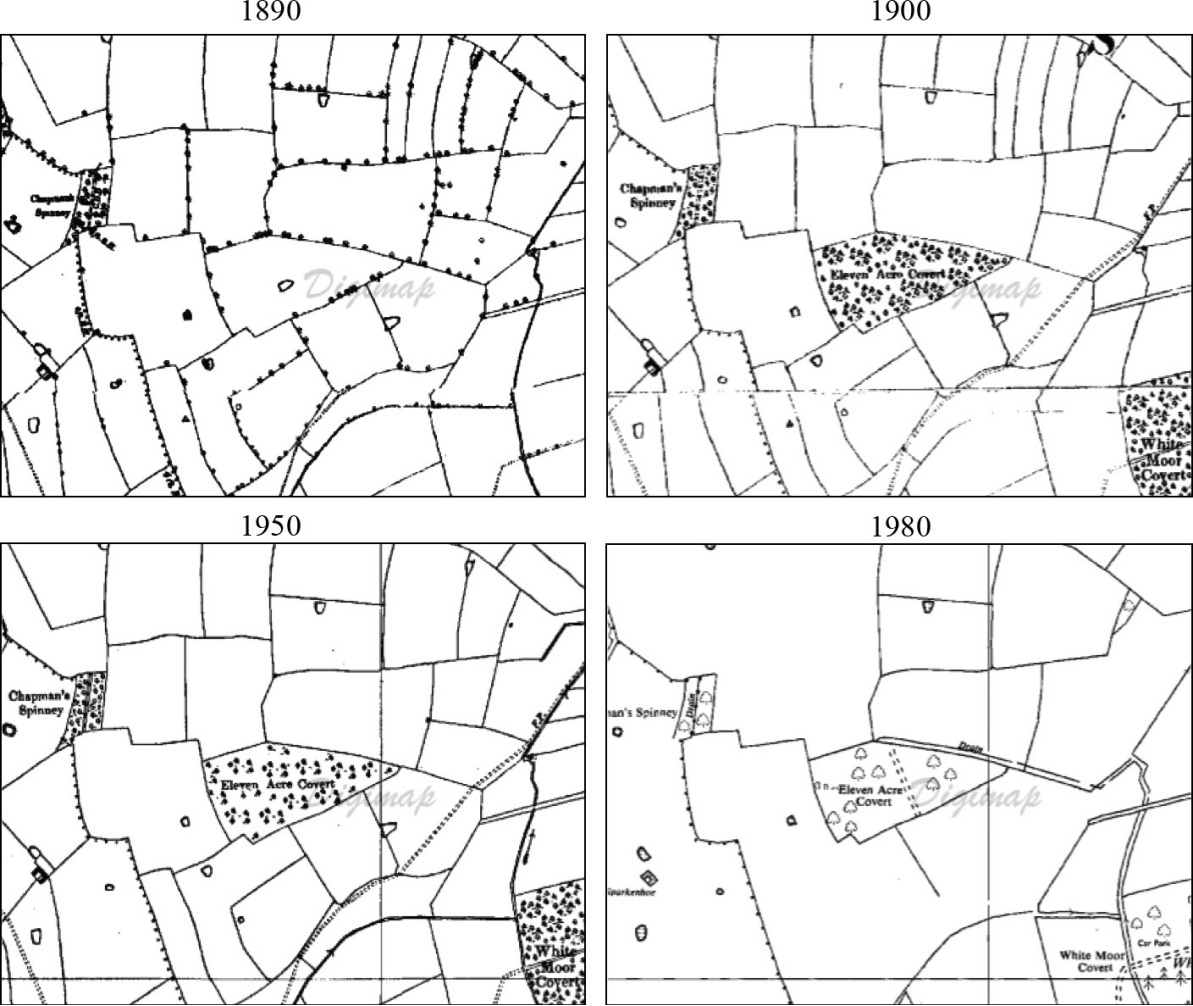
An example WrEN woodland from the England study landscapes showing the use of historical mapping to establish the age of woodland creation. The woodland labeled as “Eleven Acre Covert” appeared on the maps between 1890 and 1900, making it approximately 115 years old (EDINA © Crown Copyright 2014. An Ordnance Survey/EDINA supplied service http://digimap.edina.ac.uk/).

Four of the variables above were used as criteria for selecting a shortlist of sites for field study: (i) *patch area/size*; (iii) *site age/ecological continuity*; (iv) *amount of surrounding habitat*; and (v) *degree of spatial isolation*. This balanced the need to identify sites with combinations of a broad range of variables and the need for an approach that was amenable to desk‐based analysis, given the very large number of woodland sites in the study areas that could be considered. We used a GIS‐based site selection process based on the following steps:

1. We identified spatially discrete (i.e., not joining or forming part of another woodland) native broadleaved woodlands (>80% broadleaved canopy cover) from national woodland GIS data sets (National Forest Inventory – Forestry Commission, 2012).
2. We excluded any sites that were classified as ancient (i.e., pre‐1750s in Scotland or pre‐1600 in England) on GIS data sets of ancient woodland (Forestry Commission, 2011). This was to ensure a focus on secondary woodlands that had been planted on agricultural land and whose biodiversity would be the result of subsequent colonization rather than relict populations.
3. Within ArcGIS Desktop 10 (Advanced license, http://www.esri.com/), we measured the first three selection criteria: (i) *patch area/size*; (iv) *amount of surrounding habitat* (proportion cover of broadleaved woodland with a 3‐km buffer); and (v) *degree of spatial isolation* (measured as the distance to the nearest neighboring broadleaved woodland). For each, we calculated data quartiles that were subsequently used to ensure the final selection of sites captured the widest possible range of variables.
4. We iteratively selected suitable sites in GIS until we achieved a good spread across all the data quartiles for the three variables in step 3. This ensured all possible combinations (e.g., first quartile for (i) *patch area/size*, third quartile for (iv) *amount of surrounding habitat*, and fourth quartile for (v) *degree of spatial isolation*) were filled where possible.
5. The fourth selection criterion, (iii) *ecological continuity/age*, was manually calculated from a visual interpretation of digital scans of Ordnance Survey historical land‐use maps from 1840s to 1990s (An Ordnance Survey/EDINA supplied service http://digimap.edina.ac.uk/). We calculated the approximate age of each woodland patch by identifying the time period when the woodland was first shown on the historical maps (Fig. 2). Only woodland patches that clearly “appeared” on open agricultural land within the range of historical mapping (from 1840 to 1990) were subsequently selected. Woodlands that were present on the oldest maps, or changed shape within the mapping period, were rejected as their age could not be determined, and another site was selected from step 4.
6. We also utilized a list of more recently established woodland sites provided by the Woodland Trust, repeating the steps above to select suitable sites to add to the selection.
7. We ensured that the selected woodland sites were spatially independent, with the vast majority being at least 3 km apart (our largest scale of measurement). We also used a correlation matrix to check for potential collinearity between the main four site selection variables.

The remaining two variables (of the six identified by Humphrey et al. 2015) were measured through data collection in the field and additional spatial analysis. To measure (ii) *patch characteristics/quality*, we carried out field surveys of sites in Scotland in 2013 and 2014 and sites in England in 2014. Measurements were made of tree species richness and tree density, as part of a survey of a wider set of site characteristics. The *nature of the surrounding matrix* (vi) was investigated through spatial analysis that measured, among other things, the percentage coverage of different land covers (e.g., agriculture, seminatural vegetation, urban, water bodies) using Land Cover Map 2007 (Morton et al. 2011) within a 3‐km buffer around each site.

The data collection outlined above thus gave us data for all sites for all six broad site and landscape variables. We developed these into a series of more specific explanatory variables (Table 1) for use in subsequent analyses. We also conducted principal component analyses (PCA) to identify the most important variables driving variation between sites in each of the two study areas, at both site and landscape levels (a comprehensive list of variables included in each PCA based on Table 1 is shown in Table S1). All variables were scaled to standardize the weights of components (Jongman et al. 1995).

**Table 1:**
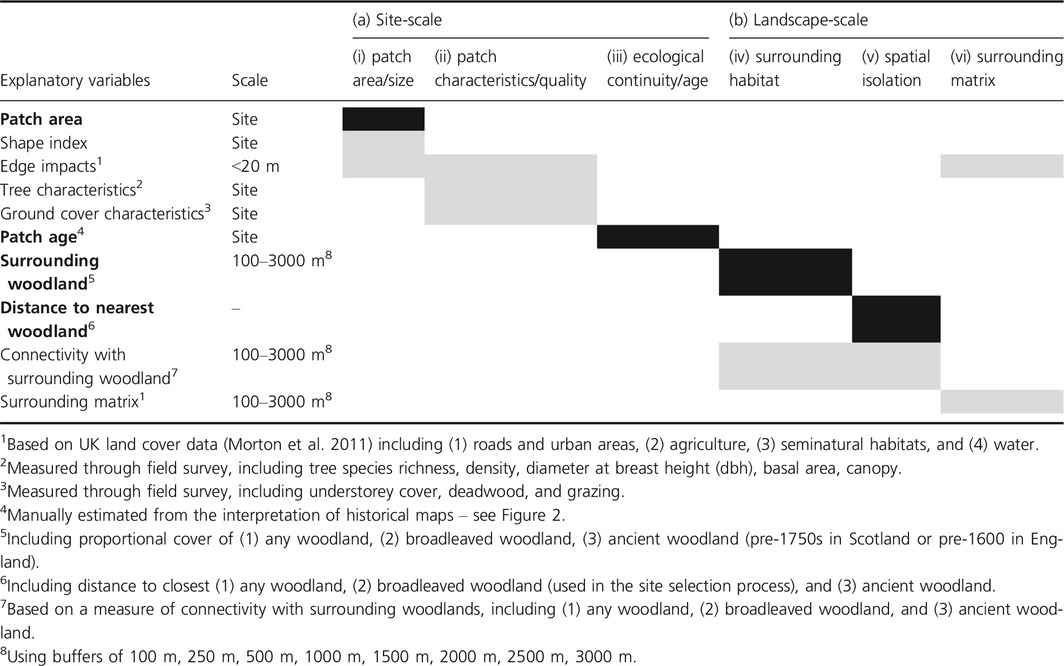
The six broad (a) site and (b) landscape‐scale variables shaping biodiversity within fragmented forest landscapes identified in Humphrey et al. (2015), and the detailed explanatory variables that were derived from these. The four variables used for site selection are indicated by black shading ‘and bold text’; gray shading indicates metrics measured in the field or using GIS data relating to those variables. Details of methods used for calculating the variables are given in footnotes

## Results

To date, we have identified 106 woodland sites matching the specified criteria within the two study landscapes (Scotland *n* = 67 and England *n* = 39) (Fig. 1). These sites range from 0.5 to 32 ha in size, are between 10 and 160 years old, are surrounded by less than 1% to 17% broadleaved woodland within a 3‐km buffer, and are between 7 and 1573 m away from the nearest broadleaved woodland (Fig. 1). The correlation matrix of the four key site selection criteria for the 106 selected sites revealed one small (Pearson correlation coefficient −0.28) but statistically significant negative correlation between (i) patch area/size and (iii) ecological continuity/age, indicating that older sites tended to be the smaller ones. No other correlations were statistically significant.

**Figure 3:**
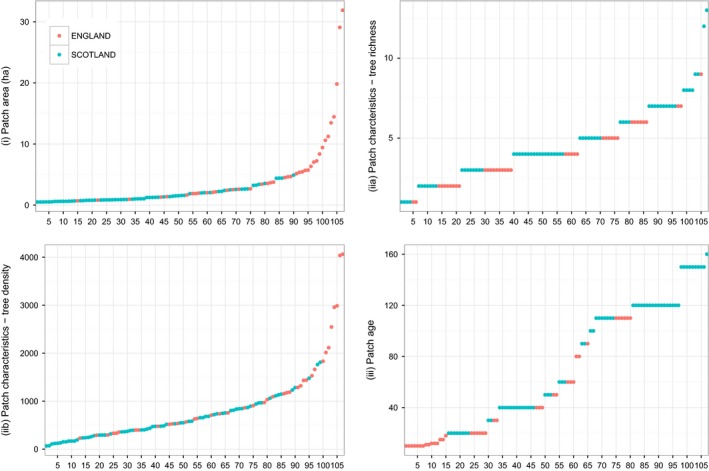
Distribution (rank order) of the four site‐scale variables: (i) patch area; (iia) patch characteristics – tree species richness; (iib) patch characteristics – tree density; and (iii) patch age in years since creation for the 106 WrEN woodland sites in Scotland (*n* = 67) and England (*n* = 39).

**Figure 4:**
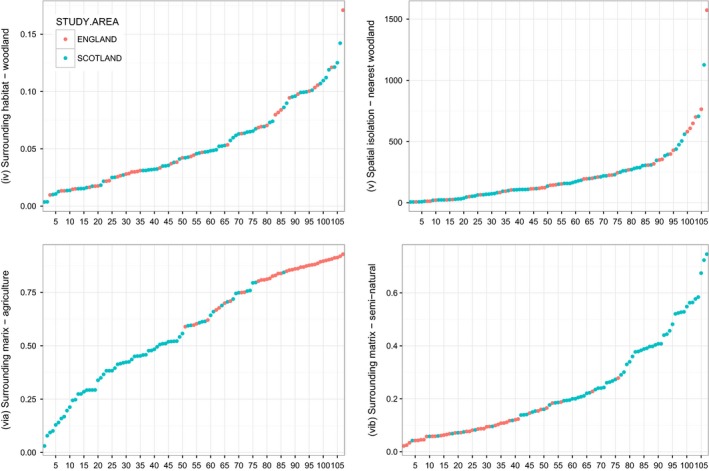
Distribution (rank order) of the four landscape‐scale variables: (iv) surrounding habitat – proportional cover of woodland within a 3‐km buffer; (v) spatial isolation – distance to nearest broadleaved woodland in meters; (via) surrounding matrix – proportional cover of agricultural land within a 3‐km buffer; (vib) surrounding matrix – proportion of seminatural land cover with a 3‐km buffer for the 106 WrEN woodland sites in Scotland (*n* = 67) and England (*n* = 39).

Table 2 presents further details of the site‐ and landscape‐level variables for the study sites, while Figures 3 and 4 show the distribution of the site and landscape variables, respectively. Figure 3 shows the general prevalence for the creation of small woodlands (mean size of 3.3 ha, SE = 0.5 – Table 2) and the tendency for older (mean 86 years, SE = 5.6), smaller (mean 1.5 ha, SE = 0.1) woodlands to occur in Scotland, in contrast to larger (mean 6.4 ha, SE = 1.1), younger (mean 42 years, SE = 5.8) woodlands in England. Figure 4 clearly shows the difference in the surrounding matrix for sites in Scotland and England, with England having a lower proportion of seminatural land cover within the surrounding landscape (mean 10%, SE = 0.01) and a far higher proportion of agriculture (mean 81%, SE = 0.02).

**Table 2:** Summary statistics for (a) four site‐scale variables: (i) patch area (ha); (iia) patch characteristics – tree species richness; (iib) patch characteristics – tree density (no. per hectare); (iii) patch age in years since creation and (b) four landscape‐scale variables; (iv) surrounding habitat – proportional cover of woodland within a 3‐km buffer (0–1); (v) spatial isolation – distance to nearest broadleaved woodland (m); (via) surrounding matrix – proportion of agricultural land within a 3‐km buffer; and (vib) surrounding matrix – proportion of seminatural habitat with a 3‐km buffer for the 106 WrEN woodland sites in Scotland (*n* = 67) and England (*n* = 39)

Explanatory variables	(a) Site‐scale				(b) Landscape‐scale			
	(i) patch area/size	(iia) patch characteristics	(iib) patch characteristics	(iii) ecological continuity/age	(iv) surrounding habitat	(v) spatial isolation	(via) surrounding matrix	(vib) surrounding matrix
	Patch area	Tree richness	Tree density	Patch age	Woodland within 3 km	Nearest woodland	Agriculture within 3 km	Seminatural within 3 km
All sites								
Min	0.5	1.0	67.0	10.0	0.00	6.7	0.03	0.02
Max	31.9	13.0	4063.0	160.0	0.17	1573.1	0.93	0.75
Mean	3.3	4.5	815.5	70.1	0.05	215.4	0.58	0.23
Median	1.8	4.0	605.5	55.0	0.05	153.2	0.60	0.19
SD	4.9	2.3	734.8	47.6	0.04	234.5	0.25	0.17
SE	0.5	0.2	71.4	4.6	0.00	22.8	0.02	0.02
Scotland								
Min	0.5	1.0	67.0	20.0	0.00	6.7	0.03	0.04
Max	4.9	13.0	1811.0	160.0	0.14	1126.4	0.84	0.75
Mean	1.5	4.8	586.0	86.1	0.05	185.0	0.44	0.31
Median	1.0	4.0	486.0	110.0	0.05	136.3	0.45	0.26
SD	1.1	2.5	397.8	46.2	0.03	188.6	0.20	0.17
SE	0.1	0.3	48.6	5.6	0.00	23.0	0.02	0.02
England								
Min	0.7	1.0	230.0	10.0	0.01	7.2	0.59	0.02
Max	31.9	9.0	4063.0	110.0	0.17	1573.1	0.93	0.28
Mean	6.4	3.9	1209.7	42.5	0.05	267.5	0.81	0.10
Median	4.5	3.0	906.0	20.0	0.04	194.6	0.85	0.09
SD	7.1	1.8	982.2	36.3	0.04	292.8	0.10	0.06
SE	1.1	0.3	157.3	5.8	0.01	46.9	0.02	0.01

In general terms, tree species richness (1–13 species), patch age (ranging from 20 to 160 years), and the amounts of agriculture (3–84%) and seminatural land cover (4–75%) in the surrounding landscape are more variable for sites in Scotland than sites in England. Patch area (0.7–31.9 ha), tree density (230–4063 trees per ha), and distance to nearest woodland (7–1573 m) are more variable in England. The variation of broadleaved woodland within the surrounding landscape is similar within both study areas, ranging from less than 1% to 14% in Scotland and 17% in England.

**Figure 5:**
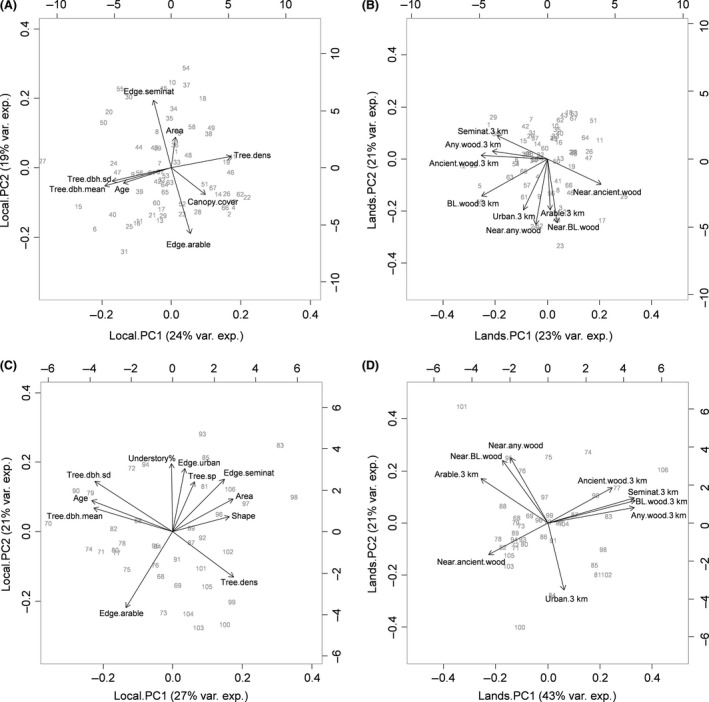
PCA biplots showing local‐level variables of WrEN sites in Scotland (A) and England (C) and landscape‐level variables in Scotland (B) and England (D). Variables with PC loadings <0.25 are not shown.

The PCA results (Fig. 5) further highlighted similarities and differences between the two study areas. They showed that the main attributes driving variation at the local scale (for both study areas) are patch age, tree diameter at breast height (dbh), and tree density (Fig. 5A and C), indicating that older woodlands generally have larger trees and lower tree densities. The amount of edge surrounded by agricultural land and the amount surrounded by seminatural vegetation were negatively correlated; this was also important in driving variation between sites in both areas. In England (Fig. 5C), the amount of urban edge, percentage of understorey cover, and tree species richness (positively correlated with each other) were also important variables. At the landscape level (Fig. 5B and D), the proportion of any woodland, broadleaved and ancient woodland, seminatural vegetation (all positively correlated with each other), and distance to nearest ancient woodland (negatively correlated with all others) were the most important variables driving variation in both Scotland and England. Distance to any woodland and broadleaved woodland, and amount of agricultural and urban areas (all positively correlated) were also important variables in Scotland (Fig. 5B). This relation was different in England, where distance to nearest woodland (particularly any and broadleaved) was positively correlated with amount of agricultural land and negatively correlated with amount of urban areas (Fig. 5D).

## Discussion

The results presented above demonstrate the potential of using historical woodland creation to construct a long‐term, large‐scale natural experiment to quantify the relative effects of site‐ and landscape‐level attributes on biodiversity within fragmented landscapes. The WrEN project clearly meets the first four of the five key design principles for a natural experiment to successfully inform landscape‐scale conservation: (1) It is focussed on habitat restoration and creation; (2) it is based on real landscapes at spatially realistic scales; (3) it covers a long time period, measuring the effects of up to 160 years of woodland development on the sites studied; and (4) the selected study sites cover a wide range of patch and landscape‐level attributes.

### Species studies

The fifth experimental design principle that we proposed above is to examine the response of a wide range of taxa. To address this, multiple species surveys have been conducted and are currently underway within the study sites. Taxa have been selected to include species with different life history traits (e.g., habitat specificity and dispersal abilities) and with populations that are likely to respond to changes in the structure, management, and spatial configuration of woodlands and their surrounding landscape at different spatial and temporal scales (see Humphrey et al. (2015). These include (i) vascular plants, (ii) lower plants (lichens and bryophytes), (iii) ground‐dwelling invertebrates (carabid beetles and spiders), (iv) flying invertebrates (Diptera and Hymenoptera), (v) small terrestrial mammals, (vi) bats, and (vii) birds.

### Analysis

In studies such as this, where there is a large number of potential explanatory variables, there can often be collinearity between variables. There is also a chance of type I statistical errors unless variables of key interest are clearly outlined a priori. In WrEN, we are interested in the relative effects of site‐ vs. landscape‐level variables on biodiversity and in predicting the likely outcome of different conservation actions, rather than hypothesis testing; thus, the effect size and amount of variation explained by each variable considered (or groups of variables, e.g., those related to local habitat characteristics vs. landscape context) are of primary importance. A useful analytical framework for these sorts of questions is provided by structural equation modeling (SEM) (Grace et al. 2010), which has been used to investigate the effects of habitat loss and fragmentation owing to its capacity to evaluate complex systems composed of nonindependent variables with direct and indirect relationships (Jamoneau et al. 2011; Brudvig et al. 2015). In addition, to examine the relative biodiversity value of these secondary woodlands, we are interested in comparing them with a number of ancient woodland reference sites.

### Challenges of natural experimentation

To date, the WrEN project has identified sites that enable sampling of a wide range of relevant site‐ and landscape‐level attributes. The study has attempted to maximize the number of study sites (currently *n* = 106) in the hope of having sufficient power to detect the desired effects. We nevertheless have some combinations of attributes represented by relatively few sites; in particular, it would have been useful to find some older and larger sites to complement the existing selection (Fig. 3). While a more even distribution of sites across all of the desired site and landscape variables would be ideal, this is one of the challenges of natural experimentation, reflecting the limited control over a study design. Another inevitable limitation of the approach (which is likely to affect not just natural experiments but also manipulative experiments conducted at large scales in real landscapes) is the effect of external factors. Within this study, we aimed to control many potentially confounding covariates by selecting sites within fairly homogeneous lowland agricultural landscapes. However, we recognize that there will be inevitable variation which is beyond our control (e.g., changes in land cover/land‐use in the surrounding landscape and variation in soil, air and water quality). This lack of complete experimental control will always be a feature of studies of this sort, but – when care has been taken in choice of study sites, as it has been here – should be more than compensated for by the spatial and temporal scales over which data can be collected. In this way, natural experiments should be viewed as complementary to approaches currently being used by other researchers, falling somewhere between experimental (controlled, less realistic) and observational (realistic, less controlled) studies (Diamond 1986; Carpenter et al. 1995; Haddad 2012).

### Benefits of historical mapping

The approach outlined in this paper is reliant on the availability of historical maps that enabled us to piece together past changes in land cover. While we acknowledge that such data are not available for all areas over sufficiently long time periods and at the necessary resolution, we note that an increasing number of studies use a diverse range of information sources to infer past land‐use. These include studies employing historical maps (e.g., De Frenne et al. 2011; Ewers et al. 2013), paleoecology (e.g., Chambers et al. 2013; Gillson 2015), aerial photographs (e.g., Surasinghe and Baldwin 2014), and documentation that may be held in museum or other collections (e.g., photographs; Sparks 2007). The potential of remote sensing to map the spatial configuration of land cover and quantify change for biodiversity research is only now starting to be realized (Corbane et al. 2015) and could provide information on land‐use changes back to 1970s. Thus, there is a variety of ways in which past land‐use and vegetation cover can be elucidated, which researchers could use as the basis for developing large‐scale natural experiments.

### Implications for future conservation

The WrEN project was initiated by a consortium of policy makers, practitioners and academics as a response to the limited and equivocal evidence base for landscape‐scale conservation, which we felt was limiting effective conservation action on the ground. The results obtained from the project will help to develop detailed recommendations to inform landscape‐scale conservation, including the design of ecological networks. These will have direct application to conservation in the UK, where ecological networks have a high priority in conservation policy and practice (Lawton et al. 2010; Defra 2011). Applications include agri‐environment schemes and the many large‐scale conservation projects (Macgregor et al. 2012) under way, as well as the management of individual protected areas.

We hope the temporal, spatial, and biological breadth of the study will also enable broad conclusions that will be relevant to conservation of forest and woodland biodiversity in similar landscapes across the world. More generally, as outlined above, we hope that the natural experimental approach we are testing here will be adopted more widely, and across a wider range of landscapes and ecosystems, to provide the evidence needed to support conservation efforts.

## Conflict of Interest

None declared.

## Supporting information


**Table S1**. PCA Summaries.
